# Detection of Zika Virus in Oropharyngeal Swabs from Patients with Acute Febrile Illness

**DOI:** 10.4269/ajtmh.22-0292

**Published:** 2022-11-07

**Authors:** Steev Loyola, Dina Popuche, Zonia Rios, Alfredo Huaman, Julia S. Ampuero, Carolina Guevara

**Affiliations:** ^1^ Grupo de Investigación UNIMOL, Facultad de Medicina, Universidad de Cartagena, Cartagena de Indias, Colombia;; ^2^ Doctorado en Medicina Tropical, Facultad de Medicina, Universidad de Cartagena, Cartagena de Indias, Colombia;; ^3^ U.S. Naval Medical Research Unit No. 6, Lima, Peru

## Abstract

The isolation of Zika virus (ZIKV) from serum of suspected human cases for diagnostic purposes can be challenging due to infrastructure constraints of laboratory testing technology. Therefore, as an alternative method, the objective of this study was to evaluate a random sample of oropharyngeal swabs for the diagnosis of ZIKV infection among patients with symptoms of arboviral and respiratory illness. The results revealed that ZIKV RNA could be detected by a reverse transcriptase polymerase chain reaction (RT-PCR) assay and isolated from oropharyngeal swabs from five of 38 samples, but serum samples from the same patients were negative for ZIKV by a variety of laboratory diagnostic approaches including RT-PCR and viral isolation followed by immunofluorescence assays. The findings suggested that the molecular detection and isolation of ZIKV in oropharyngeal swab warrants further study for consideration as an improved diagnostic procedure.

Zika virus (ZIKV) is an emerging mosquito-borne flavivirus primarily transmitted by *Aedes* mosquitoes. Since its discovery and isolation in 1947 from a sentinel macaque in Uganda, human cases of ZIKV infection have sporadically occurred in some African and Asian countries.^1^ However, it was not until 2015 that ZIKV was declared a global health emergency due to its epidemic spread in the Americas, the Caribbean, and Africa.^1^

Individuals presenting with ZIKV infections are primarily asymptomatic, but severe manifestations can occur in adults, newborns, or fetuses of infected pregnant women.^1^ Fatal cases associated with ZIKV infections were mostly reported among fetuses, immunosuppressed patients, and those with comorbidities.^1^ ZIKV diagnosis mainly relies on the detection of viral nucleic acids in the serum, detection of IgM antibodies, or, rarely, by viral isolation conducted by specialized laboratories.^2^ However, diagnosis can be challenging due to the nonspecific clinical manifestation and technical and infrastructure constraints of laboratory assays.

ZIKV has been detected in a variety of human body fluids, including serum, saliva, and others, at different periods after during the viral infection.^3^ To our knowledge, the evidence of ZIKV RNA in pharyngeal swabs is limited to two human cases: one bitten by an infected monkey^4^ and the other a Canadian traveler.^5^ Recent experimental studies suggested that ZIKV replicates and produces infectious particles in human’s upper respiratory cells^6^ and that the oropharynx and oral mucosal were potential routes of infection.^7^ Currently, the use of pharyngeal swabs is not part of recommended sampling for the laboratory diagnosis of ZIKV infection. However, these findings suggested that pharyngeal swabs could be used as a complementary sample in suspected cases of ZIKV infection with negative laboratory results in other frequently used specimens like serum. Here, we reported the assessment of oropharyngeal swabs (OS) as complementary samples for ZIKV diagnosis in cases with acute febrile illnesses and with overlapping symptoms of both arboviral and respiratory disease.

The first confirmed cases of ZIKV in Peru were reported in 2016, and the highest prevalence in the Amazon.^8^ An acute febrile surveillance study, conducted from January 1, 2016, to December 31, 2018, in dengue-endemic regions and the Amazon basin of Peru collected serum and OS from 258 subjects presenting with fever for up to 5 days plus clinical symptoms consistent with arboviral and respiratory. Of the 258 subjects, 38 (14.7%) were randomly selected out of the total subjects surveyed per study year. Specifically, of the subjects enrolled in 2016, 2017, and 2018, a total of 22 (14.9%), nine (14.3%), and seven (14.9%) were included in this study, respectively. Fever, headache, and malaise or body aches were the most frequent symptoms (38/38, 100.0%), followed by pharyngeal congestion (19/25, 76.0%), sore throat (28/38, 73.7%), cough (28/38, 73.7%), rhinorrhea (26/38, 68.4%), sputum production (22/38, 57.9%), conjunctival injection (14/25, 56.0%), rash (18/38, 47.4%), itchy throat (16/37, 43.2%), and respiratory distress (5/38, 13.2%). On average, serum and OS were collected 2.1 days (SD = 1.2) after the onset of acute febrile illness symptoms.

Acute serum samples were screened for ZIKV using a variety of diagnostic tools. Briefly, the CDC Trioplex real-time reverse transcriptase polymerase chain reaction (RT-PCR) assay was used to detect the presence of dengue virus (DENV), chikungunya (CHIKV), and ZIKV.^9^ Furthermore, ZIKV or other flavivirus replication was assessed by immunofluorescence assays (IFA) using either an anti-flavivirus monoclonal antibody (MoAb) 4G2 (Invitrogen, Waltham, MA; MA5-24387) or an in-house produced polyclonal flavivirus pool^10^^,^^11^ (yellow fever virus and DENV serotype 3) following in vitro infections in African green monkey (*Chlorocebus* species) kidney epithelial (Vero-76) cells (ATCC, Manassas, VA; CRL-1587) and in *Aedes albopictus* clone C6/36 cells (ATCC; CRL-1660). Laboratory procedures were performed as previously described.^9^^,^^10^^,^^12^^,^^13^ DENV serotype 2 was detected by RT-PCR, IFA, or both in 15 (39.5%) of the 38 serum samples tested. Neither ZIKV nor CHIKV was detected by RT-PCR or viral isolation. Because negative results in sera do not conclusively rule out ZIKV infection, OS from all subjects were subsequently tested for ZIKV and other flavivirus.

OS samples were initially tested for human influenza viruses type A and B using the CDC RT-PCR.^14^ Subsequently, OS were tested in human epithelial HEp-2 cells (ATCC; CCL-23) and Vero-76 cells for IFA screening of multiple respiratory viruses, including human influenza A and B, Parainfluenza types 1, 2 and 3, adenovirus, respiratory syncytial virus, and human metapneumovirus, as previously described.^15^^,^^16^ Two swabs (5.3%) were RNA-positive for Influenza A H3N2, and no viruses were detected by IFA or RT-PCR in the other swabs. Subsequently, all OS were tested in human lung adenocarcinoma A-549 (ATCC; CCL-185) and in C6/36 cells to isolate and identify arboviruses as described elsewhere.^16^ Cultures were evaluated daily for the presence of cytopathic effect (CPE) and harvested when cells displayed >75% (3+) CPE or up to 10 days post inoculation. Slides were prepared using harvested cells from CPE-positive and –negative cultures and two IFAs were performed as previously described^13^ using the same primary antibodies 4G2 and polyclonal flavivirus pool as described and the anti-mouse IgG-FITC (Sigma-Aldrich, St. Louis, MO; F0257) as the secondary antibody. Of the 38 OS cultured in A-549 and C6/36, 5 (13.2%) were positive for ZIKV by IFA performed in C6/36, A-549, or both harvested cells (Table 1). Taking into consideration these observations, OS that were IFA-negative in both C6/36 and A-549 cells were excluded from subsequent testing.

**Table 1:** Symptoms and results of ZIKV screening in oropharyngeal swabs

Code	Symptoms	ZIKV Ct value in swab in VTM	C6/36			A-549		
			CPE (day)	IFA: 4G2/PFP	Ct value	CPE (day)	IFA: 4G2/PFP	Ct value
FPI16534	Fever, headache, congestive pharynx, sore throat, cough, rhinorrhea, sputum production, rash, and itchy throat	29.2	Negative	2+/2+	8.92	1+ (6)	1+/1+	10.1
FPY00909	Fever, headache, malaise/body aches, sore throat, cough, rhinorrhea, sputum production, rash, itchy throat, and respiratory distress	29.7	1+ (4)	2+/1+	11.12	Negative	Negative	N.P.
FPY01047	Fever, headache, malaise/body aches, sore throat, cough, sputum production, rash, itchy throat, and respiratory distress	26.8	Negative	Negative	N.P.	2+ (5)	2+/2+	N.P.
FPY01066	Fever, headache, malaise/body aches, sore throat, cough, rhinorrhea, sputum production, rash, and itchy throat	30.2	Negative	2+/3+	N.P.	1+ (4)	2+/2+	N.P.
FPY01086	Fever, headache, malaise/body aches, sore throat, cough, rhinorrhea, rash, and itchy throat	28.0	Negative	4+/3+	N.P.	3+ (6)	2+/2+	N.P.

CPE = cytopathic effect; Ct = cycle threshold; IFA = immunofluorescence assay; N.P. = not performed; PFP = polyclonal flavivirus pool; VTM = viral transport media; ZIKV = Zika virus.

The IFA was performed using MoAb-4G2 (4G2) or a PFP as primary antibodies. Ct values were estimated by a reverse transcriptase polymerase chain reaction that targets specifically ZIKV.

Interestingly, CPE was not always observed in C6/36 and A-549 cultures even when ZIKV was detected by IFA (Table 1). In addition, ZIKV was not consistently detected in either A-549 and in C6/36 cell lines (samples FPY00909 and FPY01047) (Table 1). Both MoAb-4G2 and the polyclonal flavivirus antibody pool as primary antibodies detected ZIKV by IFA in positive viral cultures. To validate the IFA results, we extracted RNA from two C6/36 culture supernatants (samples FPI16534 and FPY00909) and one A-549 supernatant (sample FPI16534) with the most sensitive results. The three viral RNA extracts were confirmed as ZIKV-positive by RT-PCR^9^ with cycle threshold (Ct) values ranging from 8.9 to 11.1 (Table 1), thus confirming the presence of ZIKV in cultures.

Due to the evidence of ZIKV in viral cultures, viral RNA was extracted from the five primary OS for further ZIKV molecular testing.^9^ As expected, all swabs were positive for ZIKV RNA by RT-PCR with Ct values ranging from 26.8 to 30.2 (Table 1). Finally, to quantify infectious particles in primary samples, plaque assays were performed. Briefly, four 10-fold dilutions were prepared from 100 µL of the OS media with Dulbecco’s modified eagle medium (Merck & Co., Rahway, NJ; D6429) supplemented with 10% fetal bovine serum (Merck & Co., Rahway, NJ; F4135), and then inoculated onto A-549 cells in duplicates. A semisolid method was conducted using 3% of carboxymethyl cellulose^17^ and plaque forming units (PFU) were counted and used for calculating the PFU per milliliter. Of the five primary samples that were ZIKV-positive by isolation and RT-PCR, infectious particles could not be quantified in four samples. Sample FPI16534 contained 345 PFU/mL. The failure to quantify the virus in all samples could be explained by a lower sensitivity of the plaque assay compared with the initial viral isolation method.

Our results strongly suggest that ZIKV can be detected from OS collected from patients with clinical symptoms suggestive of acute febrile illnesses (Table 1) that were negative for ZIKV in their acute serum samples and for multiple virus tested by a variety of laboratory diagnostic approaches. The ZIKV detection in pharyngeal swabs is consistent with previously reported cases^4^^,^^5^ and with experiments that suggest ZIKV replication and detection in oropharyngeal mucosa.^7^ Moreover, as previously suggested,^5^ the collection of pharyngeal swabs may have implications for ZIKV diagnosis because its use as an alternative sample could rule out the possibility of false-negative cases.

The current study is subject to several limitations. First, it was performed on a small sample of subjects from whom acute serum and OS were collected. Second, urine was not collected on any subjects during this study to compare ZIKV positivity rates in respiratory swabs to rates in urine. Saliva has not been reported to add any value to the diagnosis of ZIKV infection over urine,^18^^,^^19^ even though OS have not been systematically evaluated in comparison to urine or saliva. Third, because cell culture in Vero cells was performed before ZIKV infection was suspected, we did not retrospectively test for ZIKV in Vero cell cultures from these patients from whom ZIKV was isolated in C6/36 and/or A549 cells. However, none of the Vero cell cultures exhibited signs of CPE. Lastly, convalescent serum samples were not obtained to detect the true number of seroconversions in patients who exhibited negative molecular and culture-based tests in serum and OS.

The detection of infectious ZIKV particles in OS is intriguing because it may represent a mucosal transmission route; however, this study did not investigate that possibility. It is suggestive of the possibility that ZIKV transmission may not involve vector, sexual, or vertical routes in all cases. The hypothesis of ZIKV infection through mucosa was previously proposed for a patient who only had contact with the tears or sweat from a Zika case.^20^ Interestingly, experiments conducted in rhesus macaques supports the hypothesis of ZIKV transmission via the oropharyngeal mucosal route.^7^ Although the risk of mucosal transmission has been described as low, dose-dependent, and unlikely,^7^^,^^20^ the presence of infectious particles in oropharyngeal swab warrants further studies.
